# *Pseudovibrio ascidiaceicola* 5337, a marine bacterial symbiont of the ascidian gut with unusual genome features

**DOI:** 10.1128/mra.00594-25

**Published:** 2025-07-31

**Authors:** Morgan Young, Ojas Natarajan, Shen Jean Lim, Larry J. Dishaw

**Affiliations:** 1 Morsani College of Medicine, Department of Pediatrics, Children’s Research Institute, University of South Florida, St. Petersburg, Florida, USA; 2 College of Marine Science, University of South Florida - St Petersburg Campus 92562, St. Petersburg, Florida, USA; Portland State University, Portland, Oregon, USA

**Keywords:** gram-negative bacteria, lysogeny, prophage, ascidian model, Ciona robusta, host-microbe interactions, microbiome.

## Abstract

We isolated *Pseudovibrio ascidiaceicola* strain 5337, a gut bacterium of the ascidian *Ciona robusta,* from Mission Bay, San Diego. The genomic assembly is 6.94 Mb and 99.99% complete, comprising 22 contigs and 6,613 protein-coding genes. Unicycler identified seven circular contigs, and PHASTEST identified 11 prophage regions, including two gene transfer agents.

## ANNOUNCEMENT

We isolated *Pseudovibrio ascidiaceicola* strain 5337 to explore bacterial and phage diversity and trans-kingdom interactions within the tunicate gut. Its genome suggests potential roles of prophages and horizontal gene transfer in introducing novel genetic features that contribute to the development of symbiotic relationships with marine invertebrates (1, 2).

This strain was cultured from the gut of starved *Ciona robusta*, harvested from Mission Bay, San Diego (32.780167°N, −117.242833°W) in March of 2015 (3). Pooled gut homogenates from five animals were filtered through a 0.45 µM Sterivex filter. From the filtrate, 100 µL of serially diluted aliquots were plated onto BD Difco Marine Agar 2216. Distinct isolated colonies were inoculated in BD Difco Marine Broth overnight at room temperature (20–22°C) with continuous shaking at 120 rpm (3). DNA was extracted from 1 mL of this broth (OD_600 _≥ 1.2) using the Purelink Microbiome DNA Extraction Kit (Invitrogen).

For Illumina sequencing, the DNA was sonicated, assessed on a BioAnalyzer 2100 (Agilent Technologies), size-selected to obtain fragments 400–600 bp, and used for library preparation with the NuGEN UltraLow DNA kit (Tecan Life Sciences). This library was sequenced by Eurofins MWG Operon LLC on the Illumina MiSeq 2 × 250 bp platform.

For PacBio sequencing, DNA was sheared using the Covaris g-TUBE, followed by Exo III and VII digestion, BluePippin size selection, damage repair, end repair, and adaptor ligation. DNA is purified at each step using 0.45X AMPure XP beads. Two libraries were constructed using the SMRTbell Library Preparation, 1.0 SPv3 kit and assessed using PicoGreen (Invitrogen), Agilent, and NanoDrop (Thermo Fisher Scientific) assays. Both libraries were sequenced by the University of Minnesota Genomic Center on the Sequel system using two 1M v3 SMRT cells.

On the KBase server (4), 2,765,034 Illumina-sequenced reads were trimmed using Trimmomatic v0.39 (5), and 300,239 PacBio reads (N_50 _= 9,239 bp) were trimmed to retain reads >1,000 bp using Filtlong v0.2.1 (https://github.com/rrwick/Filtlong). Default software parameters were used. Trimming retained 1,545,622 Illumina paired-end reads and 37,693 PacBio reads. Read quality was evaluated using FastQC v0.12.1 (https://www.bioinformatics.babraham.ac.uk/projects/fastqc/), while read and genome N_50_ and GC content were predicted by QUAST (Galaxy v5.3.0 + galaxy0) (6) available on the Galaxy server (7, 8).

Trimmed Illumina and PacBio reads were assembled using Unicycler v0.4.8 (9) and binned using MetaBAT2 (Galaxy v2.17 + galaxy0) (10). GTDB-Tk (Galaxy v2.4.0 + galaxy1) (11) assigned the binned assembly to the species *Pseudovibrio ascidiaceicola* with 96.86% ANI to GCF_900114245.1. The 16S rRNA sequence matched that of *Pseudovibrio* sp. FO-BEG1 (CP003147.1), with blastn comparison showing 100% query coverage and 98.79% identity (12).

The draft genome is 6.94 Mb and contains 22 contigs with 51.13% GC content and N_50_ value of 699,393 bp. The genome coverage was 3.4 × for Illumina reads and 72 × for PacBio reads. The genome is 99.99% complete with 0.57% contamination and 6,613 coding sequences, as predicted by CheckM2 (Galaxy v1.0.2 + galaxy1) (13). The genome was annotated using RAST (14, 15), Prokka (Galaxy v1.14.6 + galaxy1) (16), PADLOC v2.0.0 (17), antiSMASH v7.0 (18), Pharokka (Galaxy v1.3.2 + galaxy0) (19), the PHASTEST servers (20), and PGAP (21). PHASTEST revealed 11 prophage regions (Table 1).

**TABLE 1:** Features annotated in each contig of the *Pseudovibrio ascidiaceicola* strain 5337 genome^*b*^^,^^*c*^

Contig accession	Length (bp)	Features
JBOCEA010000001.1	1,386,464	214943:235296–prophage 1038288:1057987–terpene-precursor production 1304912:1326762–betalactone production 1337972:1370918–prophage
JBOCEA010000002.1	916,547	320644:340598–terpene-precursor production 344620:390576–prophage 774671:775024–non-ribosomal peptide synthetase-like production
JBOCEA010000003.1	765,036	135265:149158–prophage (matches Pseudovibrio sp. FO-BEG1, complete genome) (gene transfer agent) 725209:757053–non-ribosomal peptide synthetase-independent, IucA/IucC-like siderophore production
JBOCEA010000004.1	699,393	20673:51518–prophage (gene transfer agent) 33620:53840–homoserine lactone production 499543:505245–unspecified ribosomally synthesized and post-translationally modified peptide product production
JBOCEA010000005.1 ^*a*^	512,900	392766:452586–N-acyl amino acid production
JBOCEA010000006.1 ^*a*^	434,424	63737:91072–type I polyketide synthase production 91396:126547–type III polyketide synthase production 384999:396841–hydrogen cyanide production
JBOCEA010000007.1	401,274	277577:297552–terpene production
JBOCEA010000008.1	358,698	4492:13280–prophage
JBOCEA010000009.1 ^*a*^	351,948	
JBOCEA010000010.1	176,086	18554:27744–unspecified ribosomally synthesized and post-translationally modified peptide product production 162854:171643–prophage
JBOCEA010000011.1	166,137	94063:116682–prophage
JBOCEA010000012.1	141,274	
JBOCEA010000013.1 ^*a*^	140,715	
JBOCEA010000014.1	129,220	92883:111389–prophage
JBOCEA010000015.1	103,963	1494:39786–prophage
JBOCEA010000016.1 ^*a*^	81,258	
JBOCEA010000017.1 ^*a*^	81,139	
JBOCEA010000018.1	46,091	
JBOCEA010000019.1	30,308	
JBOCEA010000020.1	12,744	893:10064–prophage
JBOCEA010000021.1 ^*a*^	5,857	
JBOCEA010000022.1	1,658	

^*a*^ Circular contigs, as predicted by Unicycler.

^*b*^ Prophage regions were predicted by PHASTEST and secondary metabolite production was predicted by antiSMASH. A spreadsheet of complete annotations is available at https://github.com/ldishaw/Pseudovibrio_5337/*.* The prophage region on contig JBOCEA010000003.1 (https://www.ncbi.nlm.nih.gov/nuccore/JBOCEA010000003.1?report=asn1) matches the genomic sequence of *Pseudovibrio* sp. *FO-BEG1,* with 98% query coverage and 80.86% identity. This is the only prophage region with a match that has >50% query coverage.

^*c*^ Empty cells indicate open reading frames of unknown function.

## Data Availability

Sequence data are deposited at the National Center for Biotechnology Information (NCBI) under the BioProject ID PRJNA1261084 and BioSample ID SAMN48504407. Sequenced reads are deposited in the Sequence Read Archive (SRA) under accession numbers SRX28800662 (Illumina) and SRX28800663 (PacBio). The draft assembly is deposited in GenBank under accession number JBOCEA000000000.1.
